# Mitochondrial content, oxidative, and nitrosative stress in human full-term placentas with gestational diabetes mellitus

**DOI:** 10.1186/s12958-017-0244-7

**Published:** 2017-04-04

**Authors:** Joel Ramírez-Emiliano, Martha E. Fajardo-Araujo, Ismael Zúñiga-Trujillo, Victoriano Pérez-Vázquez, Cuauhtémoc Sandoval-Salazar, Jessica K. Órnelas-Vázquez

**Affiliations:** 1 Departamento de Ciencias Médicas, Universidad de Guanajuato, León, Gto. México; 2 Departamento de Enfermería y Obstetricia, División de Ciencias de Salud e Ingenierías, Campus Celaya-Salvatierra, Universidad de Guanajuato, Celaya, Gto. México

**Keywords:** Placenta, Gestational diabetes mellitus, Oxidative and nitrative damage, Mitochondrial content

## Abstract

**Background:**

The purpose of this study was to determine the mitochondrial content, and the oxidative and nitrosative stress of the placenta in women with gestational diabetes mellitus (GDM).

**Methods:**

Full-term placentas from GDM and healthy pregnancies were collected following informed consent. The lipid peroxidation (TBARS) and oxidized protein (carbonyls) levels were determined by spectrophotometry, and 3-nitrotyrosine (3-NT), subunit IV of cytochrome oxidase (COX4), adenosine 5′-monophosphate (AMP)–activated protein kinase (AMPK) and actin were determined by western blot, whereas ATPase activity was performed by determining the adenosine triphosphate (ATP) consumption using a High-performance liquid chromatography (HPLC) system.

**Results:**

TBARS and carbonyls levels were lower in the placentas of women with GDM compared with the normal placentas (*p* < 0.001 and *p* < 0.05, respectively). Also, 3-NT/actin and AMPK/actin ratios were higher in GDM placentas than in the normal placentas (*p* = 0.03 and *p* = 0.012, respectively). Whereas COX4/actin ratio and ATPase activity were similar between GDM placentas and those controls.

**Conclusions:**

These data suggest that placentas with GDM are more protected against oxidative damage, but are more susceptible to nitrosative damage as compared to normal placentas. Moreover, the increased expression levels of AMPK in GDM placentas suggest that AMPK might have a role in maintaining the mitochondrial biogenesis at normal levels.

**Trial registration:**

HGRL28072011. Registered 28 July 2011.

## Background

Gestational diabetes mellitus (GDM) is a glucose intolerance of varying severity with onset or first recognition during pregnancy, but the implications of GDM on fetal growth and later life remains unclear. In a meta-analysis, it was described that women with a history of GDM have a greater risk of developing type 2 diabetes mellitus (T2DM) later in life [1]. For instance, in a study, a total of 843 GDM women were followed for the development of T2DM; at 2 months’ postpartum, 105 (12.5%) subjects had T2DM (early converters). Of the 738 subjects who did not have T2DM at 2 months’ postpartum, 370 (50.1%) women attended follow-up visits for more than 1 year. Of the 370, 88 (23.8%) had newly developed T2DM (late converters) [2]. The above data suggest that GDM might be implicated in the development of T2DM in later life.

Different alterations in placentas of women with GDM have been described. For instance, villous immaturity, chorangiosis, and ischemia are significantly increased in the placentas of women with GDM. The maternal and cord plasma levels of malondialdehyde (MDA) are increased, whereas vascular endothelial growth factor (VEGF) levels are decreased in the presence of villous immaturity [3]. In addition, defective insulin signaling characterized by a significant increase in insulin receptor (IR) substrate (IRS)-1 protein expression with a concurrent decrease in IRS-2, phosphatidyl-inositol-3-kinase (PI3-K) p85a and glucose transporter (GLUT)- 4 protein expression has been demonstrated in GDM placentas, compared with normal controls [4]. Later, these data were confirmed because in placenta villi derived from GDM pregnant women exhibited differentially expressed proteins that are associated with the development of insulin resistance, transplacental transportation of glucose, hyperglycemia-mediated coagulation and fibrinolysis disorders in the GDM placenta villi. Proteins identified include Annexin A2, Annexin A5 and 14-3-3 protein that were up-regulated, while Ras-related protein Rap1A was down-regulated in GDM placenta villi at both the mRNA and protein level [5].

With respect to redox status, the normal pregnancy is associated with increased oxidative stress and exaggeration of oxidative damage. It has been reported that all oxidative stress markers, including urinary 8-hydroxydeoxyguanosine (8-OHdG), plasma 8-isoprostane, total antioxidant capacity (TAC), and erythrocyte glutathione peroxidase (GPX) and superoxide dismutase (SOD) activities, are increased in the third trimester, and most of them returned to non-pregnant levels postpartum [6]. In spite of that the above, and of the fact that the GDM placenta develops insulin resistance and alterations in the transplacental transportation of glucose, data suggest that the GDM placenta may increase its antioxidant mechanisms, thereby reducing the oxidative damage compared with control placentas. It was observed that GDM placentas are characterized by increased antioxidant gene expression [Catalase (CAT) and glutathione reductase (GSR), but there was no difference in glutathione peroxidase and superoxide dismutase], and are less responsive to exogenous oxidative stress [hypoxanthine (HX)/xanthine oxidase (XO) treatment to release cytokines] than tissues obtained from normal pregnant women [7]. Furthermore, it has been described that in vitro, the release of TNF, IL-6, and IL-8 is similar in both control and GDM placentas. Whereas, in response to oxidative stress, TNF, and 8-isoprostane release and nuclear factor-kB (NF-kB) DNA-binding activity are significantly increased in normal tissues as compared with GDM placentas. Thus, placentas from women with GDM display a reduced capacity, mediated by repression of NF-kB activity, to respond to oxidative stress [8]. Together, these data suggest a protective or adaptive mechanism that prevents damage from further oxidation *in utero* as indicated by increased tissue antioxidant expression and a reduced capacity to respond to oxidative stress in placentas of GDM women. However, in that study the oxidative damage was determined only in maternal and cord plasma and it not was determined in placental tissue.

On the other hand, alterations of the mitochondrial functions in GDM placenta are thought to play a key role in the pathogenesis of the metabolic disease and its complications. Swollen mitochondria have been observed in GDM placentas [9], and expression of mitochondrial complex I and IV proteins were significantly reduced in preeclampsia placentas [10]. The mitochondrial complex IV (Cytochrome c oxidase) is an indirect indicator of mitochondrial content, which can be determined by measuring the expression levels of cytochrome c oxidase subunit 4 (COX4). Moreover, the mitochondrial DNA (mtDNA) content in peripheral blood was decreased in GDM compared with normal pregnant women (NPW), but statistical analysis failed to document any statistically significant association [11]. These data suggest that substantial alterations in placental mitochondria occur during the development of gestational diabetes mellitus. Therefore, in the present study, the objective was to analyze mitochondrial content and oxidative and nitrosative levels in human full-term placentas with gestational diabetes mellitus.

## Methods

### Patients and sample collection

All pregnant women were screened for GDM, and women participating in the normal group had a negative screen; 12 pregnant women accepted to participate in the study. Women with GDM were diagnosed if they had two or more venous plasma glucose values greater than or equal to the defined threshold levels (fasting, ≥95 mg/dL; 1 h, ≥180 mg/dL; 2 h, ≥155 mg/dL; and 3 h, ≥140 mg/dL) on a 100-g oral glucose tolerance test between 24 and 28 weeks of gestation. All women with GDM were prescribed just dietary management. Women with any adverse underlying medical condition (i.e. including asthma, preeclampsia, and pregestational diabetes) were excluded. Then, 12 full-term placentae from GDM pregnancies and 12 from healthy pregnancies were collected following informed consent. The study was approved by the Institutional Ethical Committee of the Hospital General Regional de León, México (Reg. no. HGRL28072011).

Tissues were obtained within 10 min of delivery and dissected fragments were stored at −70 °C until further processing. A placental lobule (cotyledon) was removed from the region next to umbilical cord, the basal plate and chorionic surface were removed from the cotyledon, and villous tissue was obtained from the middle cross section. Placental tissues were blunt dissected to remove visible connective tissue and calcium deposits. Placental tissue (100 mg) was homogenized in buffer (10 mM HEPES, 0.6% Nonidet p-40, 150 mM NaCl, 1 mM EDTA) containing protease inhibitors (Complete, Boehringer Mannheim, Germany). Whole protein lysates were assayed for protein concentration using BCA protein assay (Pierce Chemical Co., Rockford, IL, USA) with BSA as the reference standard.

### SDS-PAGE and western blot

SDS-PAGE and western blot were performed as previously described [12]: 30 μg of placenta lysate was separated on 10% polyacrylamide gel, and resolved proteins were transferred to nitrocellulose membrane. Molecular weights were identified by comparison with the motility of pre-stained protein standards (Precision Plus Protein Standards, Bio-Rad Laboratories). The blots were probed with antibodies (Santa Cruz Biotechnology, Inc.) at the following dilutions (3-nitrotyrosine 1:1500, COX4 1:1500 and adenosine 5′-monophosphate (AMP)–activated protein kinase (AMPK)α1/2 1:800). Blots were stripped and re-probed with β-actin (1:3000; Santa Cruz Biotechnology, Inc.) for loading control. Proteins were detected using a chemiluminescence kit per the manufacturer’s instructions (Western Lightning Plus-ECL, Perkin Elmer) and densitometry was performed on all blots to determine the density of the bands using the Image Lab 3.0 software (ChemiDoc™ XRS, Bio-Rad).

### Determination of ATPase activity

Measuring the ATPase activity is an indirect indicator of mitochondrial content. However, it does not rule out other activities that have been described in the placenta as non-gastric H+/K + ATPase [13], Na^+^/K^+^-ATPase [14], and Ca^2+^ ATPase [15].

ATPase activity was performed by determining the adenosine triphosphate (ATP) consumption using a High-performance liquid chromatography (HPLC) system. Briefly, 32 μg of placental homogenate in 200 μl of reaction buffer (20 mM Tris-base, 5 mM MgCl_2_ and 0.1 mM ATP-Tris) were incubated at 25 °C for 10 min. The samples were then centrifuged at 14 000 rpm for 10 min, and 100 μl of supernatant were injected into the HPLC system to determine the ATP concentration. The HPLC system consisted of a GBC LC-1150 pump, GBC 1650 Advanced Autosampler and GBC LC1210K UV-vis Detector. Chromatographic separation was achieved on 250 mm × 4.6 mm SGE SS Exsil Silica column 5 μm (Thermo Hypersil-Keystone) using a mobile phase of 0.1 M KH_2_PO_4_:acetonitrile:methanol (9.6:0.3:0.1, v/v/v) with a final pH 6.3. The system was operated at room temperature (23–25 °C) with a flow rate of 0.5 mL/min. The wavelength was set at 254 nm for detection of ATP, and for quantification we used an ATP (Sigma Chemicals, St. Louis, MO, USA) concentration standard curve. Detector out- put was recorded by an integrator and digitalized using the Peak Simple software EZChrome elite. Finally, the content of ATP in placentas was also determined and considered to calculate the ATPase activity, which is expressed as pmols of ATP consumed/mg prot · min.

### Measurement of lipid peroxidation

In total homogenate of placenta tissue, lipid peroxidation levels were quantified with the thiobarbituric acid-reactive substances (TBARS) assay as we previously described [16, 17].

### Measurement of oxidized protein

Oxidized proteins were also determined in the total homogenate of placenta tissue by quantification of carbonyls content as we previously described [16, 17].

### Statistical analysis

Statistical analysis was performed using the software Statistica 8 (StatSoft, Inc). Student’s *t*-test or U of Mann-Whitney was used. The results were expressed as the mean ± standard deviation, and values were considered statistically significant if *p* < 0.05.

## Results

### Anthropometric characteristics and biochemical parameters of participants

The clinical and laboratory data of pregnant women were analyzed. There were no significant differences in maternal age, gestational age at birth, maternal BMI at birth, and gain of maternal body weight between NPW and women with GDM. Table 1 shows fasting glucose concentrations were on the borderline in women with GDM compared with NPW (*p* = 0.05), but HbA1c levels were similar between both groups. There were no significant differences with respect to the levels of lipids profile between both groups.

**Table 1:** Anthropometric characteristics and biochemical parameters of patients

	NPW patients (*n* = 12)	GDM patients (*n* = 12)	*P*
Maternal age (years)	27.7 ± 5.2	28.3 ± 5	0.8
Gestational age at birth (weeks)	38.1 ± 1.1	37.3 ± 1.9	0.3
Maternal BMI (Kg/m^2^)	32.7 ± 5.2	32.7 ± 5.3	1.0
Gain of maternal body weight (kg)	12.3 ± 8.2	10.2 ± 8.2	0.5
Glucose (mg/dL)	*70 ± 7*	*84 ± 16*	*0.05*
HbA1c (%)	5.0 ± 1	4.4 ± 0.7	0.2
Triglycerides (mg/dL)	207 ± 62	244 ± 51	0.2
Cholesterol (mg/dL)	205 ± 47	199 ± 27	0.7
HDL (mg/dL)	60 ± 12	60 ± 7	0.8
LDL (mg/dL)	104 ± 38	90 ± 30	0.4
VLDL (mg/dL)	41 ± 12	49 ± 10	0.2

*NPW* normal pregnant women, *GDM* gestational diabetes mellitus, *BMI* body mass index, *HDL* high-density lipoprotein, *LDL* low-density lipoprotein, *VLDL* very-low-density lipoprotein

### Mitochondrial content in placenta

To indirectly analyze the mitochondrial content, expression levels of COX4 was determined by western blot and the ATPase activity was performed by determining ATP consumption using a HPLC system. AMPK and actin were also determined by western blot. 30 μg of placenta lysate were used in western blot analysis. Unexpectedly, we observed variations in the actin expression levels, which was corrected by determining of the COX4, AMPK, and 3-NT/actin ratios in the same nitrocellulose membrane.

**Fig. 1:**
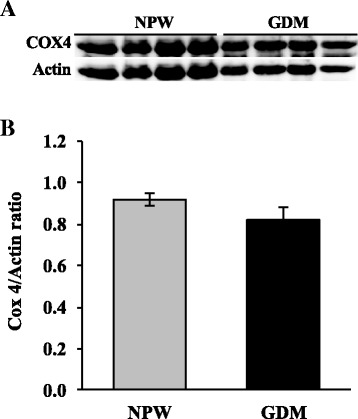
COX4 expression in placentas of GDM and healthy pregnancies. **a** Representative western blot of the COX4 and actin. **b** Densitometry analysis of the COX4/actin ratio; data are given as the means ± standard deviation (*n* = 12). NPW, normal pregnant women; GDM, gestational diabetes mellitus; COX4, subunit IV of cytochrome oxidase

**Fig. 2:**
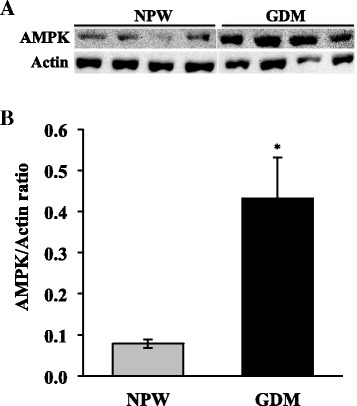
AMPK expression in placentas of GDM and healthy pregnancies. **a** Representative western blot of the AMPK and actin. **b** Densitometry analysis of the AMPK/actin ratio; data are given as the means ± standard deviation (*n* = 12). NPW, normal pregnant women; GDM, gestational diabetes mellitus; AMPK, adenosine 5′-monophosphate (AMP)–activated protein kinase. *p = 0.012 vs. NPW

**Fig. 3:**
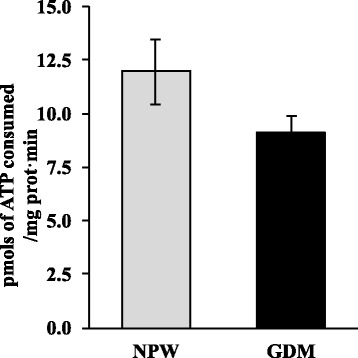
ATPase activity in placentas of GDM and healthy pregnancies. NPW, normal pregnant women; GDM, gestational diabetes mellitus. Data are given as the means ± standard deviation (*n* = 12)

COX4/actin ratios were similar between placentas with GDM and those controls (0.8 ± 0.05 and 0.9 ± 0.03, respectively) (Fig. 1a and b). Moreover, placentas with GDM did have higher AMPK/actin ratio when compared with normal placentas (0.43 ± 0.09 vs. 0.07 ± 0.01, respectively; *p* = 0.012) (Fig. 2a and b). ATPase activity was similar between placentas with GDM and those controls (9.1 ± 0.78 and 11.9 ± 1.5 pmols of ATP consumed/mg prot.min) (Fig. 3).

### Oxidative and nitrative damage in placentas

**Fig. 4:**
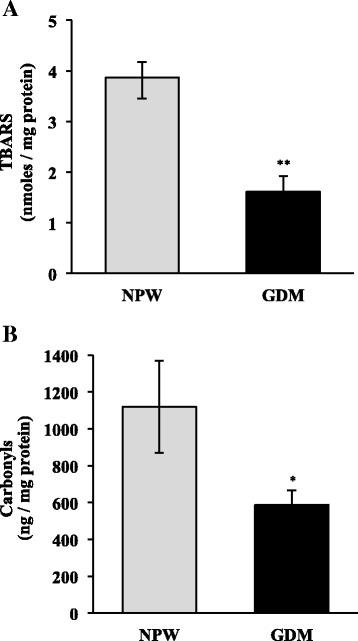
Oxidative damage in placentas of GDM and healthy pregnancies. **a** TBARS and **b** carbonyls levels. NPW, normal pregnant women; GDM, gestational diabetes mellitus. Data are given as the means ± standard deviation (*n* = 12). *p < 0.05 vs. NPW; **p < 0.001 vs. NPW

In placentas with GDM, TBARS levels were decreased as compared to normal placentas (1.6 ± 0.31 vs. 3.8 ± 0.41 nmoles/mg protein, respectively; *p* < 0.001) (Fig. 4a); moreover, in placentas with GDM, carbonyl levels were lower than in normal placentas (586.4 ± 80 and 1119 ± 249.5 ng/mg protein, respectively; *p* < 0.05) (Fig. 4b).

**Fig. 5:**
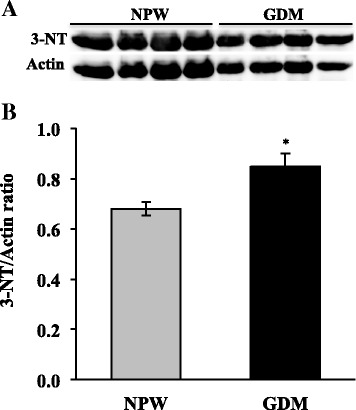
Nitrative damage in placentas of GDM and healthy pregnancies. **a** Representative western blot of the 3-nitrotyrosine (3-NT) and actin. **b** Densitometry analysis of the 3-NT/actin ratio; data are given as the means ± standard deviation (*n* = 12). ANPW, normal pregnant women; GDM, gestational diabetes mellitus. *p = 0.03 vs. NPW

With respect to nitrative damage, levels of nitrated proteins were determined by measuring the 3-nitrotyrosine (3-NT) content by western blot as is shown in the Fig. 5a; then, the 3-NT/actin ratios were determined (Fig. 5b). We found that in placentas with GDM, the 3-NT/actin ratios were higher than in normal placentas (0.85 ± 0.05 vs. 0.68 ± 0.02, respectively; *p* = 0.03).

## Discussion

It has been well established that women with GDM have higher levels of blood glucose than the NPW [3, 18]. Interestingly, in the present study, the women who were diagnosed with GDM received strict dietary control, thus at birth the fasting glucose concentrations were on the borderline of being different in women with GDM compared with NPW (70 ± 7 vs. 84 ± 16, *p* = 0.05), whereas other biochemical parameters as HbA1c and lipids profile were similar between both groups. The present data suggest that dietary control may be important to improve the biochemical alterations caused by GDM.

The present data show that the placentas of women with GDM display lower levels of oxidized lipids and proteins compared with these of NPW, suggesting a protective or adaptive mechanism that prevent oxidative damage produced during development of GDM. It is consistent with previous reports where the TBARS levels were similar between the GDM and NPW placentas [19], and protein carbonyl formation was lower in the placentas of overweight patients compared to lean individuals [20], and more importantly, catalase and glutathione reductase (GSR) mRNA expression was higher in women with GDM compared with NPW [7]. Moreover, the TBARS and SOD concentrations were similar between the placentas from GDM and NPW [21]. SOD enzyme expression and activity were also similar between placentas of the non-diabetic lean, overweight, and obese patients [20]. However, our results contrast with previous observations where the 8-isoprostane (8-IP) and protein carbonyl levels were significantly greater in GDM placentas compared to normal placentas (1720 ± 721 vs. 738 ± 177 pg/mg protein, *p* < 0.001; 0.148 ± 0.153 vs. 0.062 ± 0.011 nmol/mg protein, *p* < 0.004, respectively) [22]. Likely the discrepancy is because these authors found elevated levels of glucose prior to parturition, while the present results show that our patients had better metabolic control as was demonstrated by the levels of fasting plasma glucose and HbA1c.

Together, the findings previously reported and our results, suggest that good dietary control may be important to prevent the increased oxidative damage caused by GDM.

With respect to nitrative stress, the present results show that GDM placentas have higher levels of nitration compared with the placentas of the NPW. It is supported by a study where nitration levels were increased in the placentas of obese patients compared to lean and overweight groups; thus, with increasing maternal body mass index, there is an increase in placental nitrative stress [20]. Nitration levels of p38 MAPK were increased in placentas of preeclamptic women concomitant with a reduced catalytic activity of this protein [23]. Later, immunohistochemistry analysis demonstrated strong expression of nitrotyrosine in the placental vasculature of women with pre-eclampsia [24]. Others reported higher nitrate/nitrite concentrations in placentas from GDM patients compared with normal placentas [21], and women with preeclampsia have higher nitrite levels in the umbilical circulation and significantly more intense 3-NT immunostaining in the villous vascular endothelium of the placentas [25].

Together, the present data and previous reports suggest that GDM placentas have decreased oxidative damage because nitric oxide (NO) reacts with the superoxide anion to produce peroxynitrite, reducing the oxidative stress. However, peroxynitrite may react with tyrosine residues, increasing the nitrotyrosine levels. There may be a shift in the balance between nitrative and oxidative stress, which may be a protective mechanism for the GDM placenta. However, the clinical consequences produced by the nitration of proteins in the placenta are unknown. In this regard, increased specific nitration of MMP-2 and MMP-9 were found in full-term placentas from diabetic patients, and in vitro peroxynitrite was able to increase the activity of placental matrix metalloproteinase 2 (MMP-2) and MMP-9, suggesting that peroxynitrite can nitrate and activate MMP-2 and MMP-9 in the placenta [26]. In contrast with these data, MMP-9 was decreased in GDM placentas, but nitrate/nitrite concentrations were increased [21]. Moreover, increased nitration levels of MAPK and consequently reduced catalytic activity [23], suggesting a reduction of both the GLUT4 protein expression and mitochondrial biogenesis. Others found reduced gene expression for AMPK and mTOR in GDM placentas, but these authors did not determine protein expression [19]. Therefore, in the present study mitochondria content was indirectly determined by measuring the COX4 expression and ATPase activity.

To analyze the mitochondrial content, expression levels of COX4 and AMPK were determined, and the ATPase activity was also assayed. Our data show that GDM placentas have higher AMPK levels compared with the control placentas, whereas the COX4 levels and ATPase activity are similar between both groups. It is important to take into account that the activity that we determined represents the activity of all the ATPase in the placenta, and specifically the Na+/K(+)-ATPase activity was reduced in placental syncytiotrophoblast cells of GDM patients [27]. Is has been described that AMPK is a key for signaling kinases to induce GLUT4 expression and to increase glucose uptake in muscles [28], whereas the complex IV activity has been closely associated with mitochondrial oxidative phosphorylation capacity [29]. AMPK is also important for activating macroautophagy [30] and inducing mitochondrial biogenesis [31]. Thus, the data reported here with previously reported data suggest that GDM placentas have increased expression of AMPK in order to maintain adequate mitochondrial content, but it does not suggest whether the mitochondria are functioning properly.

We found increased AMPK levels, but we do not determine how much of this kinase is nitrated and how much is active. Therefore, it is important to determine the nitration levels of AMPK and its catalytic activity and to determine mitochondrial dysfunction in GDM placentas.

## Conclusions

Our results demonstrate that placentas of women with GDM are more protected against oxidative damage as compared to normal placentas. However, placentas with GDM are more susceptible to nitrosative damage. Moreover, the expression levels of AMPK are higher in placentas with GDM than in control placentas, suggesting that AMPK could be involved in maintaining mitochondrial biogenesis at normal levels. It is important to determine the levels of the anti-oxidant system and mitochondrial dysfunction in placentas with GDM.

## Abbreviations

3-NT: 3-nitrotyrosine
AMP: Adenosine 5′-monophosphate
AMPK: AMP–activated protein kinase
ATP: Adenosine triphosphate
BMI: Body mass index
CAT: Catalase
COX4: Subunit IV of cytochrome oxidase
GDM: Gestational diabetes mellitus
GLUT4: Glucose transporter-4
GPX: Glutathione peroxidase
HX: Hypoxanthine
IR: Insulin receptor
IRS: IR-substrate protein-1
MMP-2: Placental matrix metalloproteinase 2
NF-kB: Nuclear factor-kB
NO: Nitric oxide
NPW: Normal pregnant women
SOD: Superoxide dismutase
T2DM: Type 2 diabetes mellitus
TAC: Total antioxidant capacity
TBARS: Thiobarbituric acid-reactive substances
XO: Xanthine oxidase
